# Multiple myeloma with clivus involvement, neurological symptoms, and 45 g/L proteinorachia

**DOI:** 10.1002/ccr3.2404

**Published:** 2019-09-30

**Authors:** Yannick Chantran, Jean‐Rémi Lavillegrand, Bertrand Lefrere, Antoine Gaillet, Pierre Aucouturier, Laurent Garderet

**Affiliations:** ^1^ Immunology Department Hôpital Saint‐Antoine, AP‐HP Paris France; ^2^ INSERM UMR_S 938 Immune System & Neuroinflammation Sorbonne Université Paris France; ^3^ Service de Réanimation Médicale Hôpital Saint‐Antoine AP‐HP Paris France; ^4^ UMR_S 938 Proliferation and Differentiation of Stem Cells INSERM Paris France; ^5^ Département d'Hématologie Hôpital Pitié Salpêtrière, AP‐HP Paris France; ^6^ Sorbonne Université Paris France

**Keywords:** central nervous system, cerebrospinal fluid, hyperproteinorachia, multiple myeloma

## Abstract

The mechanical obstruction of cerebrospinal fluid (CSF) flow can lead to very high hyperproteinorachia. In myeloma, tumoral clivus obstruction can cause such high proteinorachia, associated with paraprotein detection in the CSF in absence of intrathecal synthesis.

## Graphical abstract

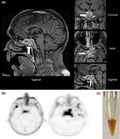

## 1 INTRODUCTION

Neurological symptoms in multiple myeloma may evoke either hypercalcemia, central nervous system (CNS) infection, or CNS involvement. We describe a myeloma patient with impairment of consciousness, associated with profoundly increased proteinorachia without signs of meningitis. We suspect this was related to mechanical obstruction caused by tumoral clivus infiltration rather than paraprotein intrathecal synthesis.

The presence of neurological symptoms in a patient relapsing after multiple myeloma (MM) may evoke either hypercalcemia, CNS infection, or MM with CNS involvement (CNS MM). The latter is rare: Approximately 1% of MM patients develop CNS MM. It is associated with poor prognosis, life expectancy in this case being 4‐6 months, thus requires rapid clinical intervention. The standard criterion for the diagnosis of CNS MM is based on cytology, which lacks sensitivity.^1^ Here we describe a patient with impairment of consciousness with a huge hyperproteinorachia but no signs of meningitis.

## 2 CASE PRESENTATION

A 64‐year‐old man with kappa free light chain MM was admitted to the intensive care unit of a tertiary referral hospital in February 2018 for acute dyspnea, fever, and confusion.

Diagnosis of MM was made in 2015, when he presented multiple lytic bone lesions, without renal failure, and 23% bone marrow plasmacytosis. The patient had an international scoring system (ISS) staging of 1. Cytogenetic was not studied at time of diagnosis, but would later reveal no high‐risk cytogenetic features (no t(4‐14) nor del17p). He received initially a lenalidomide‐dexamethasone‐ixazomib induction regimen during four cycles, followed by high dose melphalan at 200 mg/m^2^ and autologous stem cell transplantation 48 hours later. He presented pneumonia and sepsis 2 weeks after stem cell transplantation. He received a consolidation regimen 3 months after (two cycles of lenalidomide‐dexamethasone) then maintenance treatment by lenalidomide during 6 months. The patient showed partial response to treatment according to the international response criteria. The patient also displayed thrombocytopenia related to bone marrow plasma cell infiltration and drug toxicity. Iterative platelet infusions did not correct thombocytopenia.

In April 2017, the patient underwent surgery for an L2 vertebra fracture. He relapsed afterward in May 2017, October 2017, and January 2018. These three successive relapses were treated with lenalidomide‐dexamethasone‐daratumumab, with pomalinomide‐dexamethasone, and with bortezomib‐adriamycin‐dexamethasone, respectively.

In February 2018, soon after the last relapse, he presented confusion during two weeks but no focal neurological features. Blood test results were as follows: hemoglobin 11 g/dL (usual values > 13 g/dL); white blood cell count 3.530/mm^3^ (usual range 2.000‐7.500 per mm^3^); platelets 11.000/mm^3^ (usual range 150.000‐450.000 per mm^3^); Ca^2+^ 1.90 mmol/L or 76 mg/L (usual range 2.20‐2.60 mmol/L); proteins 60 g/L; creatinine 126 µmol/L or 14.25 mg/L (usual range 60‐115 µmol/L); LDH 2227 IU/L; albuminemia 32 g/L; ammonium 69 µmol/L; IgG 2.37 g/L; IgA 0.78 g/L; IgM 0.08 g/L; serum kappa free light chain (FLC) 2290 mg/L; and lambda FLC 3.5 mg/L. Blood gases under 9 L O_2_ were as follows: pH 7.52; PaCO_2_ 36 mm Hg; PaO_2_ 78 mm Hg; HCO_3_^−^ 29 mmol/L; lactate 1.0 mmol/L. He soon required mechanical ventilation. A pulmonary CT scan showed bilateral pure ground‐glass opacities. All tests for infection and autoimmunity were negative. Bronchoalveolar lavage was negative. He received daily platelet transfusions, which resulted in a transient improvement. Intra‐alveolar hemorrhage was suspected.

**Figure 1:**
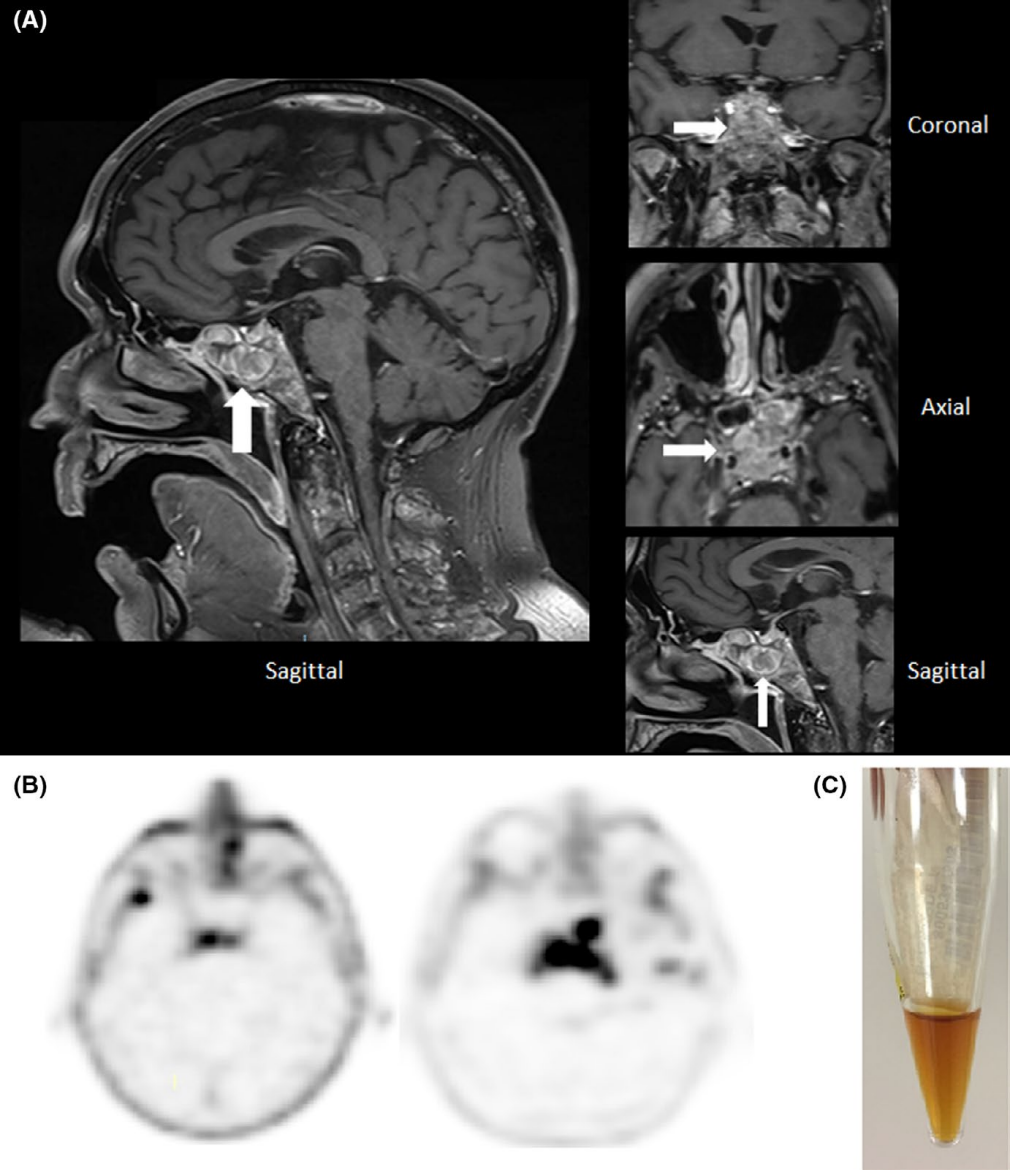
A, Cerebral MRI: sagittal T1 global sequence on the left and three dimensional T1 sequences (clivus zoom) on the right, showing a hypersignal due to an extensive process of the clivus spreading to the cavernous and sphenoidal sinuses and internal carotid sheathing. B, Choline‐PET‐CT scans showing hyperfixation of the clivus increasing between June 2017 (left) and October 2017 (right). C, Macroscopic appearance of the cerebrospinal fluid: a xantochromic honey‐like liquid similar to the serum

While recovering from pulmonary infection, the patient's neurological condition worsened, with a transient loss of consciousness. He had no history of cerebral involvement, and no lumbar puncture had been performed before. All tests for infection and autoimmunity in the cerebrospinal fluid (CSF) were negative. The EEG under propofol was normal and reactive. However, in addition to already known diffuse vertebral infiltration, cerebral and spinal MRI showed an infiltrating lesion of the clivus spreading to the cavernous and sphenoidal sinuses and internal carotid sheathing (see Figure 1, panel A). Retrospectively, hyperfixation of the clivus with increasing choline uptake between June and October 2017 was identified on the PET‐CT scan (Figure 1, panel B). The CSF obtained by lumbar puncture displayed a xantochromic honey‐like appearance, macroscopically similar to a serum sample (Figure 1, panel C). Cytocentrifugation of the CSF revealed few red blood cells but no macrophages, plasma cells or other abnormal cells. Analysis of the CSF showed: nucleated elements 0/mm^3^; RBC 1600/mm^3^; proteins 44.6 g/L (usual range 0.15‐0.30 g/L); glucose 5.6 mmol/L or 1.0 g/L; Cl^‐^ 107 mmol/L; and LDH 824 U/L. The remarkably elevated proteinorachia was further investigated, including by protein electrophoresis and immunofixation in paired serum and CSF samples. The CSF exhibited a profile similar to that of the serum with all protein fractions in their usual proportions, with a CSF/serum albumin ratio of 1.30. Given the similar electrophoretic aspect between CSF and serum, the research of oligoclonal bands in the CSF was not performed. The kappa monoclonal FLC was present in the same proportion as in the serum (see Table 1). The patient died of multiple organ failure within a few days of admission to hospital.

**Table 1:** Results of paired serum and CSF analyses

	Serum	CSF
Proteins	52 g/L	44.6 g/L
Albumin	29.4 g/L	22.5 g/L
lambda FLC	<2 mg/L	3.5 mg/L
kappa FLC	4340 mg/L	2290 mg/L
β2‐microglobulin	6.4 mg/L	6.26 mg/L
LDH	1796 U/L	824 U/L
Glucose	1.50 g/L	1.00 g/L

Abbreviations: CSF, cerebrospinal fluid; FLC, free light chain.

## 3 DISCUSSION

Extramedullary involvement is observed in about 7% of MM patients at diagnosis, while another 6% develop extramedullary lesions later in the course of the disease. Involvement of the CNS is estimated to be present in 1% of patients. The definitive diagnosis is histological, depending on the presence of atypical plasma cells in the CSF or direct tissue sampling. However, CSF cytology is poorly sensitive. An indirect diagnosis requires radiologically proven plasmacytoma arising in a location noncontiguous with the bone, that is, leptomeningeal enhancement and/or an intraparenchymal lesion.^1^, ^2^, ^3^, ^4^ In such cases, intrathecal production of the monoclonal immunoglobulin or plasma cell by‐products provides the rationale for CSF FLC measurement. This test has been reported to be more sensitive than cytology and to correlate with the response to therapy.^5^ Moreover, such intrathecal synthesis can lead to markedly elevated proteinorachia.^6^

The present case did not fit the canonical description of CNS involvement during MM: In spite of neurological symptoms that occurred at MM relapse, which strongly suggested CNS involvement, no plasma cells could be detected in the CSF. Imaging showed no leptomeningeal or parenchymal enhancement. Moreover, there was no elevation of the involved FLC or β2‐microglobulin in the CSF as compared to serum. On the other hand, cerebral MRI revealed an infiltrating lesion of the clivus spreading to the cavernous and sphenoidal sinuses and internal carotid sheathing. Another striking feature of this case is the remarkably elevated nonselective proteinorachia (45 g/L), in the absence of radiological signs of intracranial hemorrhage. Blood uptake during lumbar puncture could be ruled out since only a few red blood cells were present in the CSF sample.

Two mechanisms, not mutually exclusive, may be envisaged to explain such elevated nonselective proteinorachia: an increase in the passage of serum proteins into the CSF or a decrease in protein clearance in the CSF. Traditional views consider that the CSF is mainly produced by the choroid plexus and to a lesser extent at the blood‐brain barrier. Water, solutes, and cells then flow through the ventricular system and/or the extracellular space of the brain into the subarachnoid space, from where the CSF flows out of the CNS through the arachnoid villi into the dural venous sinuses. Sheaths around the cranial nerves provide an alternative pathway for CSF outflow, predominantly through the cribriform plate, into the lymphatic system of the nasal mucosa, draining the deep cervical lymph nodes.^7^, ^8^ The relative contributions of these two pathways remain poorly known.^9^

The most frequent interpretation of an increased protein concentration in the CSF is the so‐called “protein leakage” from the serum characterizing barrier impairment. Actually, it has been shown convincingly that a reduced CSF turnover is in most cases able to explain the presence of increased levels of blood‐derived proteins in the CSF. A reduced CSF flow may be due to a lower CSF production rate (eg, induced by carbonic anhydrase inhibitors), or a lower outflow on account of local mechanical (eg, protein complexes), inflammatory or tumoral obstruction of the CSF outflow pathways.^10^

Considering this case, it was believed at first that this patient presented intrathecal synthesis of FLC, given the very high concentration of FLC in the CSF. If the participation of such intrathecal synthesis in the elevated paraproteinorachia cannot be formally discarded in this case, it cannot either entirely explain the strikingly high and unselective proteinorachia. Rather more, impairment of the CSF outflow through the cribriform plate and cranial nerve sheaths might explain the markedly elevated proteinorachia and the neurological symptoms. In addition, although measurement of FLC in the CSF can provide useful information concerning suspected plasma cell dyscrasia with CNS involvement,^5^ this method is not yet standardized. In the absence of reference values, it requires confirmation using other analytical methods for the determination of CSF proteins such as immunofixation.^11^

## 4 CONCLUSION

Very high proteinorachia can occur in myeloma patients without meningitis. In this case, we suspect that it was related to a mechanical obstruction caused by tumoral clivus infiltration, rather than intrathecal synthesis. Clonal free light chain was increased in the CSF, but to the same extent as in the serum.

## CONFLICT OF INTEREST

The authors have no conflict of interest to declare.

## AUTHOR CONTRIBUTIONS

JRL, AG, and LG: managed the patient. YC, BL, and PA: performed the analysis. YC, JRL, PA, and LG: wrote the manuscript. All authors reviewed and approved the final version of the manuscript.
